# Assessment of functional capacity in children with chronic kidney disease using various field tests

**DOI:** 10.1007/s00467-026-07306-6

**Published:** 2026-05-11

**Authors:** Merve Firat, Aydan Asli Aksel-Uylar, Melda Saglam, Rezan Topaloglu, Fatih Ozaltin, Ali Duzova, Naciye Vardar-Yagli, Bora Gulhan

**Affiliations:** 1 Department of Physical Therapy and Rehabilitation, Kırşehir Ahi Evran University, Kırşehir, Türkiye; 2 Faculty of Physical Therapy and Rehabilitation, Hacettepe University, Ankara, Türkiye; 3 Faculty of Medicine, Department of Pediatrics, Division of Pediatric Nephrology, Hacettepe University, Ankara, Türkiye

**Keywords:** Chronic kidney disease, Children, Functional capacity, Muscle strength, Patient preference

## Abstract

**Background:**

Children with chronic kidney disease (CKD) often have a reduced functional capacity. The study aimed to evaluate the functional capacity of children with CKD by using three field tests: 6 min walk (6MWT), 2 min walk (2MWT), and 3 min step test (3MST), and investigate relationships between these tests and to determine which tests patients prefer.

**Methods:**

This study evaluated 51 children with CKD (19 female, 32 male). The 6MWT, 2MWT, and 3MST were used to measure functional capacity. After performing the tests, participants reported their preferred one. Quadriceps muscle strength (QMS) and hand grip strength (HGS) were assessed using dynamometers.

**Results:**

The mean age of the patients was 13.7 ± 3.2 years. A total of 22 patients (43.1%) were in stage 2 CKD, 13 patients (25.5%) in stage 3, six patients (11.8%) in stage 4, two patients (3.9%) in stage 5 non-dialysis (3.9%) and eight patients (15.7%) in stage 5 on dialysis. No statistically significant differences were observed between the early and advanced CKD groups for any of the field tests (*p* > 0.05). However, the early CKD group had a significantly higher dominant QMS than the advanced CKD group (*p* = 0.023). Most children preferred walking tests (6MWT: 43.1%; 2MWT: 43.1%), while 13.8% preferred the 3MST. Moderate positive correlations were found between 6 and 2MWT (*r* = 0.669, *p* < 0.001) and between 6MWT and 3MST (*r* = 0.422, *p* = 0.002).

**Conclusions:**

Peripheral muscle strength was deteriorated in advanced CKD. The 2MWT is a practical and time-efficient alternative to the 6MWT. The 3MST remains a viable option when walking tests are not feasible.

**Trial registration:**

NCT06683339.

**Graphical abstract:**

A higher resolution version of the Graphical abstract is available as Supplementary information.

**Figure:**
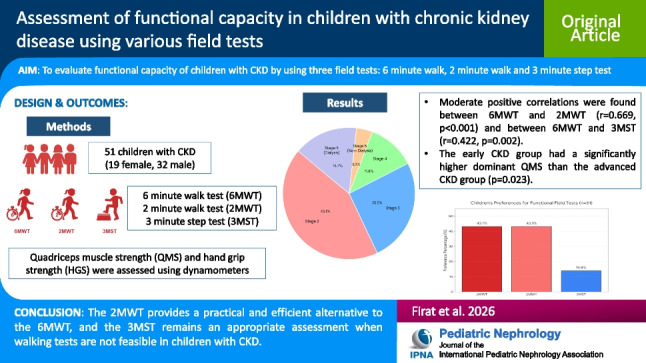

**Supplementary Information:**

The online version contains supplementary material available at 10.1007/s00467-026-07306-6.

## Introduction

Functional capacity is the ability to perform daily activities that depend on aerobic metabolism and the coordinated function of the pulmonary, cardiovascular, and skeletal muscle systems [1]. Higher cardiorespiratory fitness is linked to better metabolic and cardiovascular health in children [2]. Cardiopulmonary exercise testing, which measures maximal oxygen consumption (VO₂max), is widely used to assess functional capacity [3]. However, because most daily activities do not require maximal effort, functional capacity also encompasses the ability to perform submaximal tasks [1].

Walking is the main form of daily locomotion, requiring adequate endurance for independent function. The 6 min walk test (6MWT), recommended by the American Thoracic Society, is widely used in children. The 2 min walk test (2MWT) provides a shorter assessment, strongly correlating with 6MWT distance [4]. The 3 min step test (3MST) is another simple, equipment-free field test involving repetitive stepping [5].

Children with chronic kidney disease (CKD) show lower functional capacity compared to healthy peers [6–8]. In this group, functional capacity relates to protein-energy wasting, inflammation, glomerular filtration rate, physical activity, fat mass, and lipid levels [9, 10]. Fatigue, poor exercise performance, and low activity motivation may further exacerbate muscle loss and functional decline [7].

Research into functional capacity in pediatric CKD remains relatively limited compared with other chronic conditions. In studies conducted, the 6MWT has generally been used as a field test to assess cardiorespiratory and submaximal functional capacity [11, 12]. However, evidence for the use of shorter or alternative field tests such as the 2MWT and 3MST in this population is still sparse, underscoring the need for further research. This study aimed to assess functional capacity using different field tests, investigate their relationship with kidney functions, peripheral muscle strength, compare within-test physiological responses across the different field tests, and determine patients’ test preferences.

## Materials and methods

### Patient characteristics

This cross-sectional study was conducted at Hacettepe University, Faculty of Physical Therapy and Rehabilitation between May 2022 and June 2025. Considering the 6MWT and 3MST correlation coefficient in the pilot data, the required sample size was calculated as 46, with 90% power and 5% type 1 error (G*Power, Version 3.1.9.2, Universität Düsseldorf, Germany). The study included clinically stable patients with CKD aged 6–18 years who were being followed up at the Pediatric Nephrology Department of Hacettepe University Faculty of Medicine. Estimated glomerular filtration rate (eGFR) was calculated using the original Schwartz formula [13]. CKD was categorized as 5 stages according to KDIGO classification [14, 15]. Individuals who declined participation, as well as those with musculoskeletal, neurological, pulmonary, cardiovascular disease, cancer, and cognitive impairments likely to interfere with functional capacity assessment were excluded.

The patients’ demographic information was recorded, and their clinical information was obtained from the specialists’ medical records.

### Functional capacity

Patients’ functional capacity was assessed using 6MWT, 2MWT, and 3MST. The 6MWT was performed in a 30 m corridor according to the American Thoracic Society criteria. The individual was asked to walk as fast as they could for 6 min without running. At the end of the period, the total distance walked was recorded [16]. The 2MWT was conducted in a 15.2 m corridor, and the test instructions were the same as for the 6MWT. After the 2 min, the total walked distance was noted [4]. The 3MST was performed on a 15 cm step. The speed was set at 30 climbs and descents per minute and controlled by a metronome. After 3 min, the total number of steps was recorded [17]. Before and after the tests, heart rate, oxygen saturation, perception of dyspnea, general, and leg fatigue according to the modified Borg scale (MBS) were measured. The MBS is a 0–10 subjective rating scale used to quantify perceived exertion, with 0 indicating no exertion and 10 indicating maximal exertion [18]. These measurements were used to evaluate the physiological response elicited by each test method. Once all the tests had been performed, the patients were asked which one they preferred.

### Peripheral muscle strength

Patients’ quadriceps muscle strength (QMS) was measured using a portable handheld dynamometer (Lafayette Instrument Company, Lafayette IN, USA) [19]. Hand grip strength (HGS) was evaluated using a grip dynamometer (Jamar, Fabrication Enterprises Inc., Irvington, NY 10533, USA) [20]. The measurements were repeated three times and the highest values were recorded in kilogram-force (kgF) and analyzed.

### Statistical analysis

The statistical analysis was conducted using SPSS Statistics version 29.0. The Kolmogorov–Smirnov test was used to examine data normality. Data were given as the mean and standard deviation (SD), median (minimum–maximum), number and frequency. The relationship between functional capacity tests and peripheral muscle strength was evaluated using Pearson’s or Spearman’s correlation coefficient, according to distribution. Due to their non-normal distribution, the Kruskal–Wallis test was used to compare changes in heart rate, dyspnea, general and leg fatigue during functional capacity tests. The correlation coefficients were classified as negligible (0.0–0.19), weak (0.20–0.39), moderate (0.40–0.69), strong (0.70–0.89), and very strong (0.90–1.0) [21]. The level of significance was determined as *p* ≤ 0.05. The study protocol was approved by the Non-Interventional Clinical Research Ethics Committee of Hacettepe University (GO 22/237) and written informed consents were obtained from the parents and patients.

## Results

### Patients’ characteristics

A total of 51 patients (19 female, 32 male) were included in the analysis. The mean age of the patients was 13.7 ± 3.2 years. At the time of study, 22 patients (43.1%) were in stage 2 CKD, 13 patients (25.5%) in stage 3, six patients (11.8%) in stage 4, two patients (3.9%) in stage 5 non-dialysis (3.9%) and eight patients (15.7%) in stage 5 dialysis. The most common cause of CKD was congenital anomalies of the kidneys and urinary tract (51%). Other demographic and clinical characteristics of the patients are given in Table 1.

**Table 1:** Demographic and clinical characteristics of the patients

Characteristics	Patients (*n* = 51)
Age, years (mean ± SD)	13.7 ± 3.2
Gender (female/male), n	19 (37.3%)/32 (62.7%)
Height, cm (mean ± SD)	154.9 ± 16.3
Weight, kg (mean ± SD)	48.5 ± 17
Body mass index, kg/m^2^ (mean ± SD)	19.6 ± 4.1
eGFR, mL/min/1.73 m^2^ (mean ± SD)	52.1 ± 29.5
*CKD stage*	
Stage 2, *n*	22 (43.1%)
Stage 3, *n*	13 (25.4%)
Stage 4, *n*	6 (11.8%)
Stage 5 non-dialysis, *n*	2 (3.9%)
Stage 5 dialysis, *n*	8 (15.7%)
*CKD etiology*	
Congenital anomalies of the kidney and urinary tract, *n*	26 (51%)
Glomerular diseases, *n*	8 (15.7%)
Others, *n*	17 (33.3%)
*Tests*	
6MWT distance (m) (mean ± SD)	524.3 ± 71
2MWT distance (m) (mean ± SD)	184.9 ± 28.4
3MST step count (n) (mean ± SD)	82.1 ± 10
QMS-dominant (kgF) (mean ± SD)	19.5 ± 7.6
QMS-nondominant (kgF) (mean ± SD)	18.2 ± 6.6
HGS-dominant (kgF) (mean ± SD)	25.3 ± 9.8
HGS-nondominant (kgF) (mean ± SD)	24.2 ± 9.3

*CKD* chronic kidney disease, *eGFR* estimated glomerular filtration rate, *6MWT* 6 min walk test, *2MWT* 2 min walk test, *3MST* 3 min step test, *QMS* quadriceps muscle strength, *HGS* hand grip strength

### Relationship between eGFR and physical performance parameters

Specifically, there was no significant correlation found between eGFR and measures of functional capacity tests, including the 6MWT (*r* = 0.157, *p* = 0.270), 2MWT (*r* = 0.073, *p* = 0.612), and 3MST (*r* = −0.072, *p* = 0.618). In contrast, our results reveal statistically significant positive correlations between eGFR and peripheral muscle strength. Specifically, there was a weak association between dominant QMS and eGFR (*r* = 0.321, *p* = 0.022), as well as between dominant HGS and eGFR (*r* = 0.278, *p* = 0.048) (Table 2).

**Table 2:** Relationship between renal function and physical performance parameters

Parameters	eGFR, mL/min/1.73 m^2^
6MWT distance (m)	*r* = 0.157, *p* = 0.270
2MWT distance (m)	*r* = 0.073, *p* = 0.612
3MST step count (n)	*r* = −0.072, *p* = 0.618
QMS-dominant (kgF)	*r* = 0.321, *p* = 0.022*
QMS-nondominant (kgF)	*r* = 0.264, *p* = 0.061
HGS-dominant (kgF)	*r* = 0.278, *p* = 0.048*
HGS-nondominant (kgF)	*r* = 0.261, *p* = 0.064

*6MWT* 6 min walk test, *2MWT* 2 min walk test, *3MST* 3 min step test, *eGFR* estimated glomerular filtration rate, *QMS* Quadriceps muscle strength, *HGS* hand grip strength

### Comparison of physical performance parameters in children with early- and advanced-stage CKD

The physical performance parameters of the early (stage 2–3) CKD group (*n* = 35) were compared with those of the advanced (stage 4–5) CKD group (*n* = 16). No statistically significant differences were observed between the early and advanced CKD groups for any of the functional capacity tests (*p* > 0.05). In contrast to the functional capacity tests, a significant difference was observed in one measure of peripheral muscle strength. The early CKD group had a significantly higher dominant QMS than the advanced CKD group, and this difference was statistically significant (*p* = 0.023). The non-dominant QMS was higher in the early CKD group than in the advanced CKD group, but this difference did not reach the significance threshold (*p* = 0.058). No significant differences were observed for either the dominant or non-dominant HGS (*p* > 0.05) (Table 3).

**Table 3:** Comparison of physical performance parameters in patients with early- and advanced-stage CKD

	Early CKD (stage 2–3) (*n* = 35)	Advanced CKD (stage 4–5) (*n* = 16)	*p*
Parameters			
6MWT distance (m)	531.0 (417.7–646.4)	520.3 (238.0–648.0)	0.428
2MWT distance (m)	188.3 ± 24.2	177.4 ± 35.7	0.210
3MST step count (n)	86 (44–90)	84.50 (74–90)	0.698
QMS-dominant (kgF)	21.2 ± 7.6	16.0 ± 6.6	0.023*
QMS-nondominant (kgF)	19.4 ± 6.7	15.6 ± 5.7	0.058
HGS-dominant (kgF)	26.5 ± 9.6	22.7 ± 10.0	0.207
HGS-nondominant (kgF)	25.3 ± 9.3	22.0 ± 9.2	0.253

*6MWT* 6 min walk test, *2MWT* 2 min walk test, *3MST* 3 min step test, *QMS* quadriceps muscle strength, *HGS* hand grip strength

### Relationship between functional capacity tests and peripheral muscle strength

**Fig. 1:**
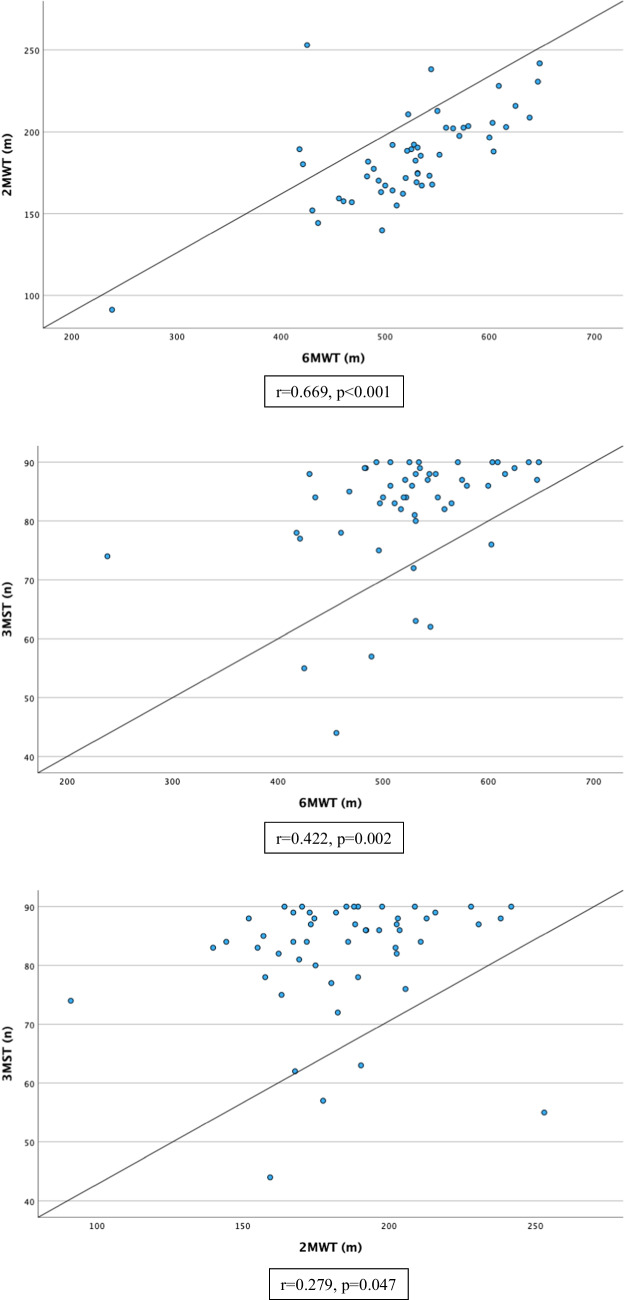
Correlations between functional capacity tests

**Fig. 2:**
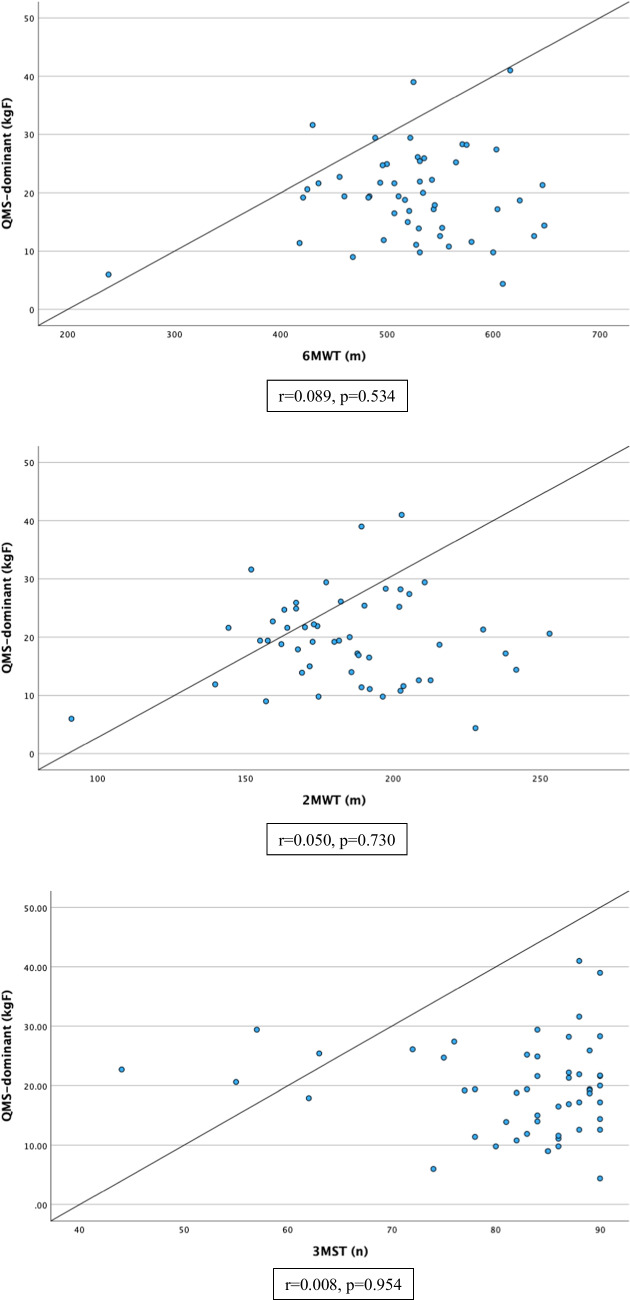
Correlations between functional capacity tests and QMS-dominant

**Fig. 3:**
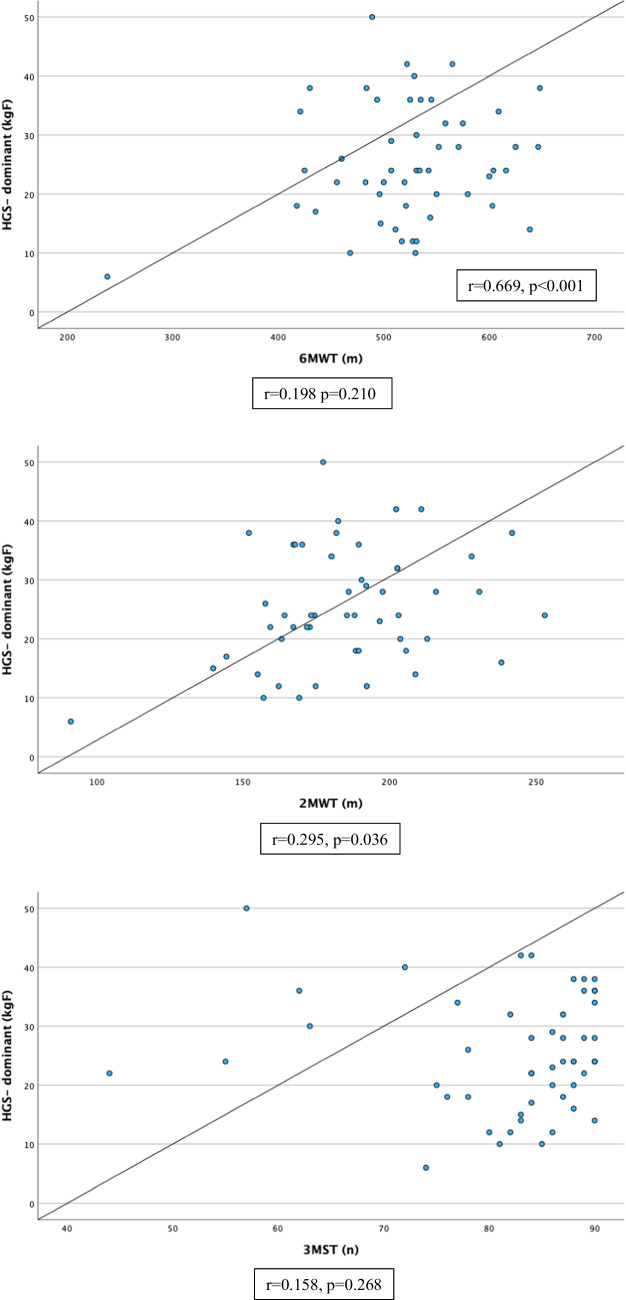
Correlations between functional capacity tests and HGS-dominant

Correlation analysis indicated a statistically significant relationship between the three functional capacity tests. Moderate positive correlations were found between 6 and 2MWT distances (*r* = 0.669, *p* < 0.001) and between 6MWT distance and 3MST step count (*r* = 0.422, *p* = 0.002). A weak positive correlation was found between 2MWT distance and 3MST step count (*r* = 0.279, *p* = 0.047) (Fig. 1). With regard to peripheral muscle strength, there were no statistically significant correlations between QMS on the dominant or non-dominant side and any of the functional capacity tests (*p* > 0.05) (Fig. 2). The HGS on the dominant side showed a weak but statistically significant correlation with 2MWT distance (*r* = 0.295, *p* = 0.036) whereas the correlations for the other HGS comparisons did not reach statistical significance (Fig. 3). When asked about their preferred functional capacity test, 43.1% of patients said they preferred the 2MWT, 43.1% said they preferred the 6MWT, and 13.8% said they preferred the 3MST.

### Change in physiological responses

All patients tolerated these tests and did not note any side effects. Increases in heart rate, oxygen saturation, dyspnea, general, and leg fatigue during functional capacity tests were observed after all three tests; however, these differences were not statistically significant (Table 4).

**Table 4:** Changes (Δ) in heart rate, oxygen saturation, perception of dyspnea, general, and leg fatigue during functional capacity tests

Parameters	6MWT distance (m)	2MWT distance (m)	3MST step count (*n*)	*p*
ΔHeart rate (beats/min) (median (range))	56 (−7, 87)	43 (8–70)	44 (10–90)	0.116
ΔOxygen saturation (SpO_2_) (median (range))	−1 (−7, 2)	−1 (−8, 2)	−1 (−6, 2)	0.708
ΔDyspnea Δ(MBS; 0–10) (median (range))	3 (0–10)	2(−4, 5)	2 (−3, 10)	0.154
ΔGeneral fatigue (MBS; 0–10) (median (range))	1 (−4, 5)	1 (−2, 5)	1 (−3, 9)	0.394
ΔLeg fatigue (MBS; 0–10) (median (range))	1 (−2, 7)	1 (−4, 5)	2 (−2, 10)	0.134

*MBS* Modified Borg scale

## Discussion

Our study enlightens a weak association between dominant QMS and eGFR as well as between dominant HGS and eGFR. Besides, there were moderate positive correlations between 6MWT, 2MWT, and 3MST step count. While all three tests are interrelated, only a weak association exists between dominant HGS and the 2MWT.

The cardiopulmonary exercise test is considered the best approach for evaluating functional capacity. However, due to the obstacles associated with cardiopulmonary exercise testing, field tests are used more frequently as they are easier to implement and less costly [22]. Field tests such as time-based walking tests are an easy, affordable, and safe method of evaluating performance [23]. The 6MWT is a widely used method of assessing functional capacity in children, including those with CKD. This six-minute test only requires a 30-m corridor and equipment for assessing vital signs. The test is easy to administer and is well tolerated by patients because it reflects a level of exercise similar to that experienced in daily life [24–28]. In our study, the mean distance achieved in the 6MWT was 524.37 ± 71.01 m, which appears to be lower than that observed in healthy children [24]. This finding is consistent with previous research suggesting that children with CKD often have reduced functional capacity [7, 8].

Although the 6MWT is widely used to assess functional capacity, it has been suggested that individuals with weaker muscles and lower exercise tolerance may struggle to complete it. Evaluating functional capacity with shorter test times may therefore be necessary. The 2MWT could be a good alternative for individuals with short attention spans who do not want to do the 6MWT [23]. The role of 2MWT in assessment of functional capacity in CKD children is limited. In the present study, the moderate correlation observed between the 6MWT and the 2MWT indicates that both tests capture a similar construct of functional exercise capacity in children with CKD. While this relationship has been demonstrated in other pediatric and adult populations, the present study is the first to report this association in children with CKD, thereby providing novel evidence supporting the use of the 2MWT in this population [23, 29–31]. This finding’s clinical implications are significant. While the 6MWT is widely used and well validated, it requires a long corridor, sufficient testing time and sustained motivation. These factors may not always be feasible in busy outpatient clinics, inpatient settings or when assessing children with fatigue. By contrast, the 2MWT can be carried out more quickly and in a smaller area, yet still provide meaningful information about functional exercise capacity. In addition, the majority of participants in the present study preferred walking-based tests, such as the 2MWT, demonstrating their feasibility and acceptability. Overall, these findings suggest that the 2MWT could be a more practical and efficient way of monitoring functional capacity in children with CKD than the 6MWT. One of the main findings of this study is that the 2MWT is a valid, applicable and acceptable alternative measure of functional capacity in children with CKD. This result aligns with and significantly expands upon the application of shorter field tests that have previously been validated for other pediatric chronic conditions, such as neuromuscular disorders and cystic fibrosis (CF) [23, 29]. These findings confirm that the 2MWT is an appropriate and effective tool for measuring limitations related to endurance caused by these diseases. A significant improvement in 2MWT distance was observed in children with CF after treatment [29]. This suggests that the 2MWT could be used as a sensitive clinical measure to monitor the current capacity and response to treatments such as exercise, nutrition, or dialysis in children with CKD. Another important point is that the relationship between the 6MWT and the 2MWT reported in our study is also supported by other studies conducted in different populations [30, 31]. Our study expands the application of the 2MWT to this patient group and highlights its potential for routine clinical assessments where time, space, or patient tolerance are limiting factors. There are several obstacles to walking tests. The 6MWT and 2MWT tests require a corridor length of 15–30 m. However, access to such a testing area may not always be available in clinical settings, and patient evaluation may require the use of tests that can be performed in smaller areas. Additionally, alternative tests may be needed for hospitalized patients who must remain in isolation rooms and for those who are unable to walk long distances and can only perform stepping in place. Therefore, the 3MST is also a test that can be considered an alternative [22]. It does not need a large area, motivation or verbal support, and it can be used outside of laboratories. The test involves ascending and descending a 15 cm step at a constant speed of 30 steps per minute for 3 min [32]. The literature shows that this test has been used safely in many pediatric populations including healthy children, those with CF and primary ciliary dyskinesia [22, 32, 33]. In the study by Firat et al., which used the same 3MST protocol, it was observed that healthy children achieved a higher step count than children with primary ciliary dyskinesia [33]. In the present study, children with CKD also demonstrated lower step counts compared with these published healthy reference data, suggesting reduced functional exercise capacity. On the other hand, the results of our study on the relationship between 6MWT and 3MST are consistent with those of other studies in the literature [5, 22]. Our results, supported by literature, suggest that the 3MST is an effective, low-cost alternative that can be incorporated into the clinical follow-up of pediatric CKD patients for the early detection of functional decline.

This study provides the first evidence of the feasibility and validity of the 3MST in children with CKD. This is an important addition to the existing literature, as no previous studies have examined the 3MST in this population. It was demonstrated that the 3MST was well tolerated and showed significant positive correlations with both the 6MWT and the 2MWT, supporting its construct validity as a measure of functional exercise capacity in pediatric CKD. These findings suggest that the 3MST could be used as an alternative method of assessing functional capacity, particularly in clinical environments where the length of the corridor, the endurance of the patient, or the attention span are limited. By confirming the association between the 3MST and walking tests, and by demonstrating the applicability of the 3MST in pediatric CKD, our study expands the current evidence base for field-based exercise testing. It also supports the integration of this simple, low-cost test into clinical follow-up for the early detection of functional decline.

Our study included children without musculoskeletal problems. However, in pediatric patients with CKD with limited mobility or wheelchair dependence, for whom walking- or stepping-based tests are not feasible, functional capacity may be assessed using alternative tests targeting the upper extremities; 6 min push test, one stroke push test, shuttle ride test and graded wheelchair propulsion test can be given as an example of these tests [34, 35].

Our study observed that children with CKD tolerated all tests well. Additionally, none of the three tests resulted in a significant increase in dyspnea, fatigue, or leg fatigue. These results confirm the safety and feasibility of these tests for children with CKD. On the other hand, our study showed that gait-based assessments (6MWT and 2MWT) had significantly stronger correlations with each other than 3MST, suggesting that tests simulating natural walking activities may more accurately reflect the daily functional abilities of children with CKD. In our study, 86.2% of patients favored either the 6MWT or 2MWT, whereas only 13.8% preferred the 3MST. The fact that walking is a more common activity for children than climbing stairs may explain why they prefer it [24].

The lack of significant differences in heart rate, oxygen saturation, and subjective results between the tests may be due to the tests being performed at a submaximal level or the children’s inexperience in rating perceived exertion [23, 32, 36]. It is anticipated that the level of perceived exertion will be elevated in the 3MST in comparison to walking tests [5, 22]. The wide variations in the results of our study, coupled with the lack of difference in perceived exertion across the tests, suggest that subjective assessments may be less reliable in this age group.

The existing literature is inconclusive regarding the relationship between peripheral muscle strength and functional capacity in children with CKD. In their study, Alaylı et al. emphasized the relationship between functional capacity assessed with the 6MWT and QMS in children undergoing peritoneal dialysis [37]. Unlike the aforementioned study, there was no significant correlation between QMS and any of the field test results in our study. There is also a study showing that there is no relationship between QMS and the 6MWT in pediatric patients on chronic hemodialysis and kidney transplant recipients, which is consistent with the results of our study [10, 38]. Although the quadriceps muscle contributes biomechanically to walking and stepping, isolated measures of quadriceps strength may not correlate with field tests such as the 6MWT, 2MWT, or 3MST, as these tests reflect integrated cardiopulmonary and neuromuscular performance rather than the function of a single muscle group [39]. In addition, QMS was assessed using an isometric dynamometric method, whereas walking and stepping tasks involve repetitive dynamic muscle contractions, which may partly explain the lack of a direct association. Furthermore, only a weak correlation was found between dominant HGS and 2MWT distance. The weak association between dominant HGS and the functional capacity test observed in our study may be due to the relatively low muscle mass of children with CKD, as well as the complex interplay of factors such as protein-energy wasting, chronic inflammation, worsened kidney function, low physical activity level and nutritional deficiencies, which affect performance independently [9, 10]. Our results showed that eGFR was not correlated with functional capacity tests (6MWT, 2MWT, and 3MST), but there were weak significant associations with peripheral muscle strength (QMS and HGS). The absence of significant correlations between kidney function and functional capacity tests, and the lack of significant differences in test performance between early- and advanced-stage CKD groups, should not be interpreted as evidence that these assessment tools are invalid. Instead, these findings probably reflect the fact that submaximal functional tests capture distinct physiological domains compared with isolated muscle strength measurements. Because functional exercise capacity reflects the integrated performance of the cardiovascular, respiratory, neuromuscular, and metabolic systems, it may remain relatively preserved in children with CKD despite decreased kidney function, or it may mask the direct effects of kidney failure [40]. This aligns with previous studies indicating that kidney dysfunction is more closely associated with muscle strength than with overall aerobic performance. Strength tends to decline early in CKD due to factors such as inflammation, metabolic acidosis, uremic toxins, and protein-energy wasting [41].

In this pediatric CKD cohort, we noted that, while functional capacity (as assessed by field tests) did not differ significantly between early-stage (stage 2–3) and advanced-stage (stage 4–5) CKD, there was a notable decline in dominant QMS in the advanced group. Several important implications for understanding musculoskeletal health in children with CKD are presented by this difference between muscle strength and functional capacity. The absence of significant differences in functional capacity tests, despite lower dominant QMS in children with advanced CKD, suggests that standard field tests may not fully capture localized muscle involvement. The way in which muscle weakness presents itself in children may affect the results of walking or general performance tests [7, 37]. A recent study in pediatric CKD reported that QMS was significantly associated with balance, underlining the importance of proximal muscle strength for fundamental activities [7]. In our study, no significant difference was found in terms of HGS between early and advanced CKD groups. This finding is supported by previous literature demonstrating that although children with CKD exhibit reduced HGS compared with healthy peers, the severity of grip strength reduction does not strongly correlate with eGFR across CKD stages. The lack of a strong correlation between CKD severity and HGS decline suggests that factors other than kidney function account for the effect of CKD on muscle strength [42]. In our study, children with advanced CKD had significantly lower dominant QMS than children with early-stage CKD, even without significant declines in commonly used functional capacity tests. This discrepancy suggests that assessing muscle strength, particularly that of the lower extremity muscles, may help to detect early musculoskeletal impairments.

One of the key strengths of this study is the evaluation of functional capacity using multiple field-based tests in a pediatric CKD population. However, some limitations have been identified. Firstly, the cross-sectional design prevents any causal inferences regarding the relationship between muscle strength and functional capacity from being made. Secondly, the study did not include a control group of healthy children. However, the values were interpreted by referencing the literature. Future studies including age- and sex-matched healthy control groups are important to establish normative comparisons and further validate these findings. As a third limitation, this study included only ambulatory children with CKD who did not have musculoskeletal problems. Therefore, the findings regarding field test preferences and feasibility cannot be generalized to pediatric patients with CKD who have limited mobility or are wheelchair-dependent. Additionally, in our hospital serum creatinine measurements were performed by Jaffe assay, which is not an enzymatic method. Therefore estimated eGFR was calculated using the original Schwartz equation.

Finally, as it was a single-center study, patients in CKD stages were not distributed evenly. Due to the fact that physical function worsens with disease severity, shorter testing times may be preferable at later stages of the disease. Further studies on this topic involving larger populations are needed.

## Conclusion

This study shows that the 6MWT, 2MWT, and 3MST are safe and feasible methods of assessing functional capacity in children with CKD. The 6MWT test is widely used in clinics. However, the 2MWT might be ideal for use in environments where resources are limited, as it is a more practical and time-efficient alternative to the 6MWT. Though children with CKD preferred the 3MST less, it could still be used to assess the functional capacity. The routine implementation of such simple, low-cost tests in clinical practice could facilitate the early detection of functional decline and enable timely interventions to preserve functional capacity in pediatric CKD patients.

## Supplementary Information

Below is the link to the electronic supplementary material.

ESM 1 Graphical abstract (PPTX 955 KB)

## Data Availability

The datasets used and/or analyzed during the current study are available from the corresponding author on reasonable request.
